# Supplementary material for: Construction of a Yeast Cell-Based Assay System to Analyze SNAP25-Targeting Botulinum Neurotoxins
Source: Microorganisms. 2023 Apr 26;11(5):1125. doi: 10.3390/microorganisms11051125 (PMC10221579; doi:10.3390/microorganisms11051125)
Supplement: Supplementary file 1 [file microorganisms-11-01125-s001.zip › microorganisms-2282368-supplementary.pdf]

Figure S1

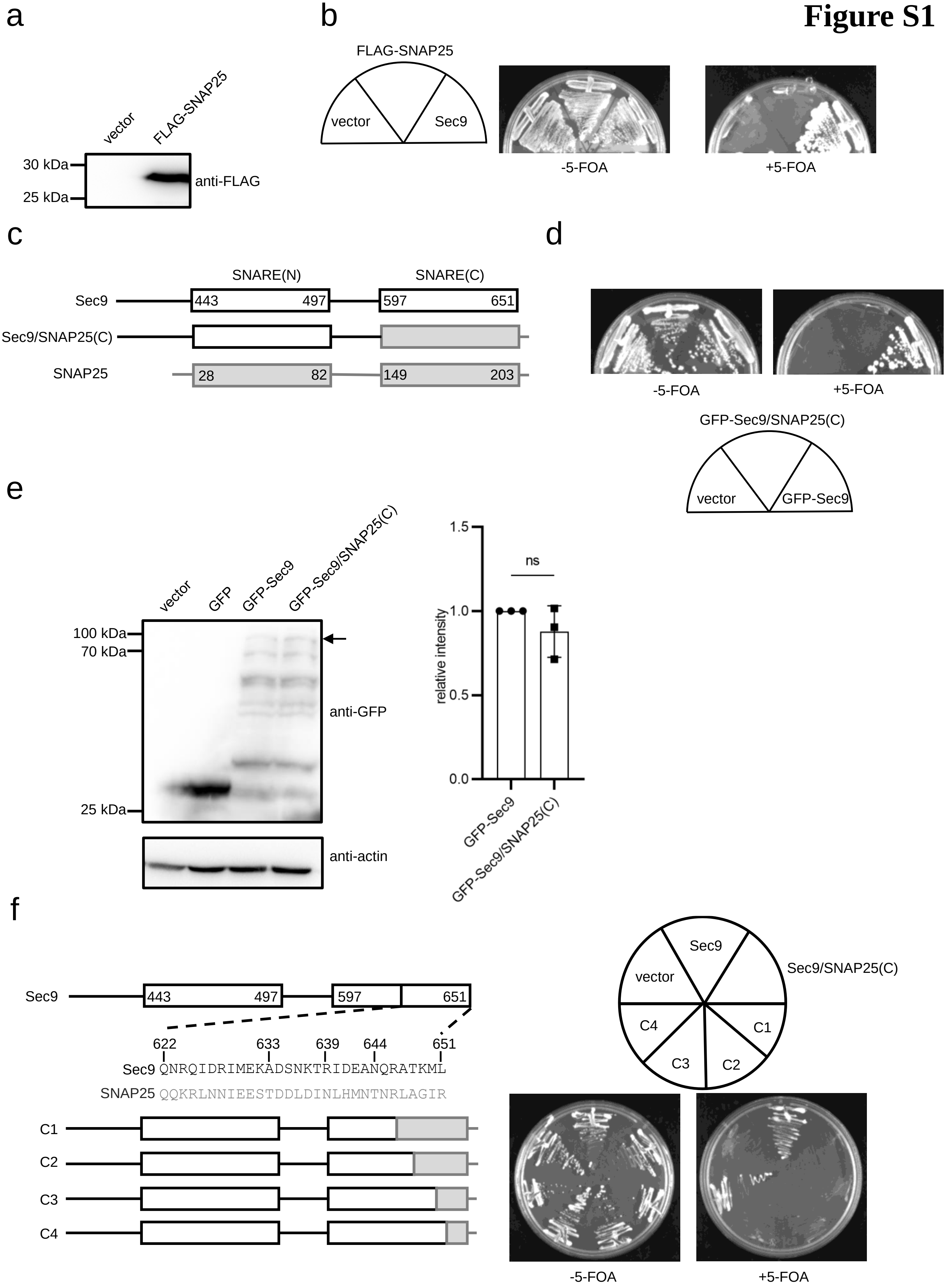

a
vector
FLAG-SNAP25
30 kDa
25 kDa
anti-FLAG
b
FLAG-SNAP25
vector
Sec9
-5-FOA
+5-FOA
c
SNARE(N)
SNARE(C)
Sec9
443
497
597
651
Sec9/SNAP25(C)
SNAP25
28
82
149
203
d
-5-FOA
+5-FOA
GFP-Sec9/SNAP25(C)
vector
GFP-Sec9
e
vector
GFP
GFP-Sec9
GFP-Sec9/SNAP25(C)
100 kDa
70 kDa
25 kDa
anti-GFP
anti-actin
ns
relative intensity
1.5
1.0
0.5
0.0
GFP-Sec9
GFP-Sec9/SNAP25(C)
f
Sec9
443
497
597
651
622
633
639
644
651
Sec9 QNRQIDRIMEKADSNKTRIDEANQRATKML
SNAP25 QQKRLNNIEESTDDLDINLHMNTNRLAGIR
C1
C2
C3
C4
Sec9
vector
Sec9/SNAP25(C)
C4
C1
C3
C2
-5-FOA
+5-FOA

Figure S1 Complementation assay of Sec9/SNAP25 chimeras.
(**a**) *sec9*Δ cells harboring pRS316TEF-SEC9 were transformed with pRS314-TEFpr (vector), or pRS314-TEFpr-FLAG-SNAP25 (FLAG-SNAP25). Their lysates were subjected to western blot analysis using an anti-FLAG antibody. (**b**) *sec9*Δ cells harboring pRS316TEF-SEC9 were transformed with pRS314-TEFpr (vector), pRS314-TEFpr-FLAG-SNAP25 (FLAG-SNAP25), or pRS314-TEFpr-SEC9 (Sec9). These cells were streaked on SD plates supplemented with or without 5-fluoroortic acid (5-FOA) to select cells lacking pRS316TEF-SEC9. (**c**) Schematic representations of Sec9, SNAP25 and Sec9/SNAP25(C). Numbers indicate amino acid positions. White and gray boxes represent SNARE domains of Sec9 and SNAP25, respectively. (**d**) *sec9*Δ cells harboring pRS316TEF-SEC9 were transformed with pRS314-TEFpr-GFP (vector), pRS314-TEFpr-GFP-SEC9 (GFP-Sec9), or pRS314-TEFpr-GFP-Sec9/SNAP25(C) (GFP-Sec9/SNAP25(C)), and they were streaked on SD plates supplemented with or without 5-FOA. (**e**) Left panel: Lysates of yeast cells used in (**d**) were subjected to western blot analysis using anti-GFP and -actin antibodies. Right panel: Relative intensities between GFP-Sec9 and GFP-Sec9/SNAP25(C). Intensities of their bands were normalized to actin. Intensity of GFP-Sec9 band was defined as 1. Data are presented as the mean ± SEM. Statistical significance was determined by two-tailed unpaired Student's *t* tests. n=3. ns, not significant ($P \geq 0.05$). (**f**) Left top panel: Alignment of the latter half of the SNARE(C) domains of Sec9 and SNAP25. Left bottom panels: Schematic representation of Sec9/SNAP25 chimeras termed C1 to C4. White and gray boxes represent regions derived from Sec9 and SNAP25, respectively. Numbers indicate amino acid positions in Sec9. Right panel: *sec9*Δ cells harboring pRS316TEF-SEC9 were transformed with pRS314-TEFpr (vector), pRS314-TEFpr-SEC9 (Sec9), pRS314-TEFpr-Sec9/SNAP25(C) (Sec9/SNAP25(C)), pRS314-TEFpr-C1 (C1), pRS314-TEFpr-C2 (C2), pRS314-TEFpr-C3 (C3), pRS314-TEFpr-C4 (C4) and they were streaked on SD plates supplemented with or without 5-FOA.

Figure S2

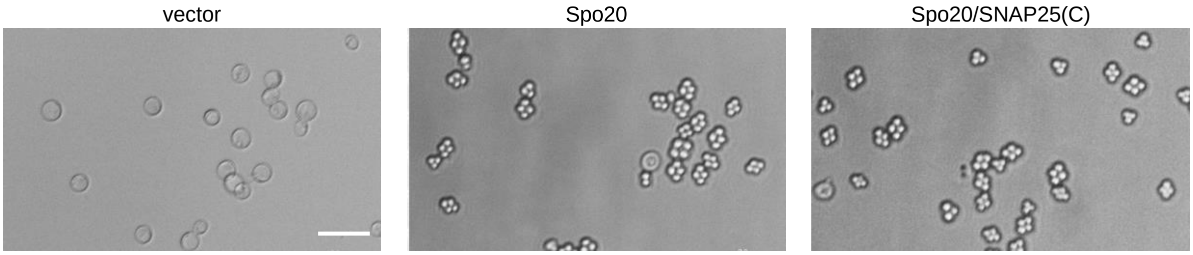

Figure S2 Complementation assay of Spo20/SNAP25(C) chimera.
*spo20*Δ cells transformed with pRS306-SPO20pr (vector), pRS306-SPO20pr-HA-Spo20 (Spo20) or pRS306-SPO20pr-HA-Spo20/SNAP25(C) (Spo20/SNAP25(C)) were cultured in sporulation media for 48 h and observed by bright-field microscopy. Representative images are shown. Bar, 20 µm.

Figure S3

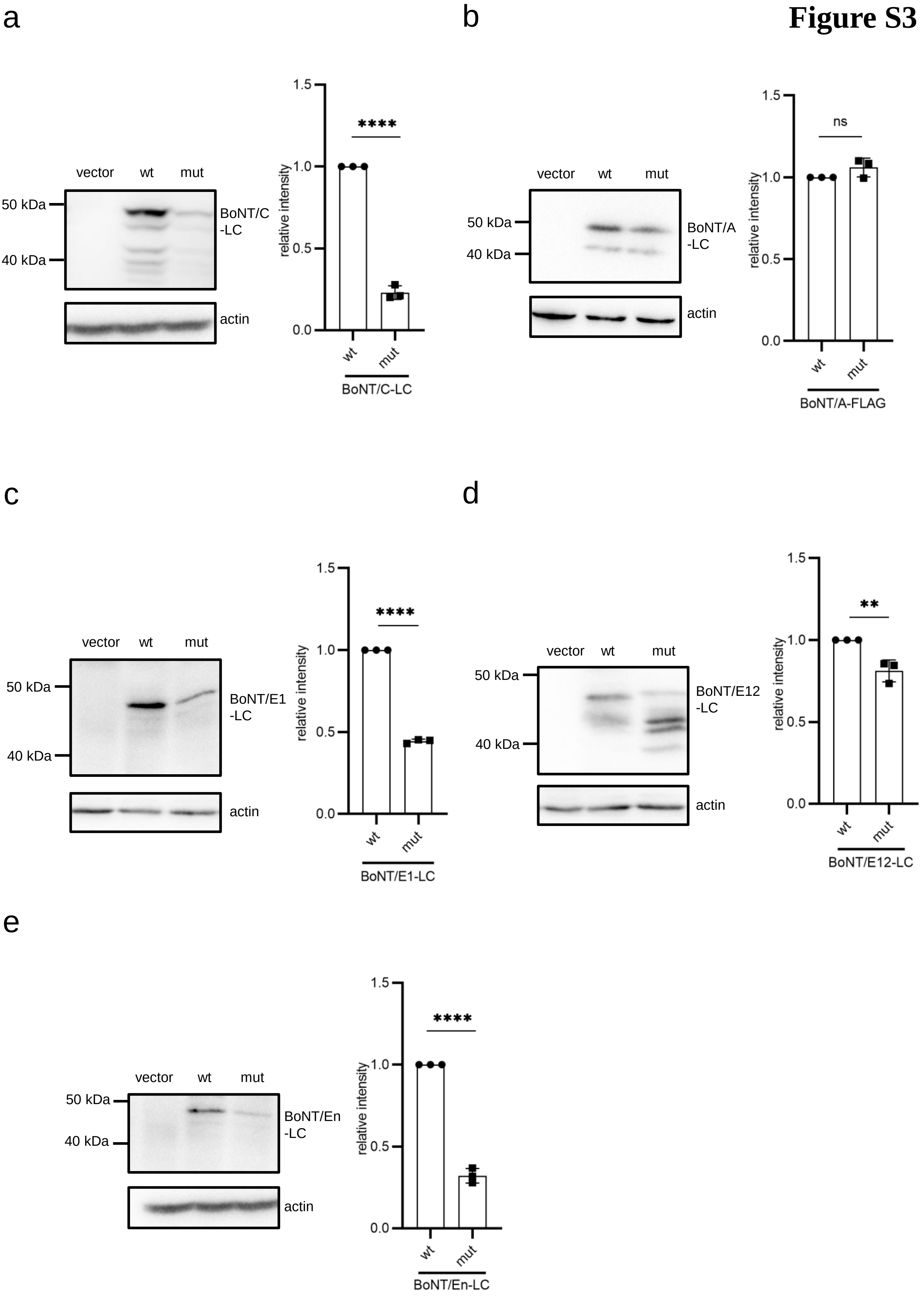

a
vector
wt
mut
50 kDa
40 kDa
BoNT/C
-LC
actin
relative intensity
1.5
1.0
0.5
0.0
****
wt
mut
BoNT/C-LC
b
vector
wt
mut
50 kDa
40 kDa
BoNT/A
-LC
actin
relative intensity
1.5
1.0
0.5
0.0
ns
wt
mut
BoNT/A-FLAG
c
vector
wt
mut
50 kDa
40 kDa
BoNT/E1
-LC
actin
relative intensity
1.5
1.0
0.5
0.0
****
wt
mut
BoNT/E1-LC
d
vector
wt
mut
50 kDa
40 kDa
BoNT/E12
-LC
actin
relative intensity
1.5
1.0
0.5
0.0
**
wt
mut
BoNT/E12-LC
e
vector
wt
mut
50 kDa
40 kDa
BoNT/En
-LC
actin
relative intensity
1.5
1.0
0.5
0.0
****
wt
mut
BoNT/En-LC

Figure S3 Expression levels of wild-type and mutant BoNT-LCs.
(**a**) *spo20*Δ cells harboring HA-Spo20/SNAP25(C) were transformed with pRS424-TEFpr (vector), pRS424-TEFpr-BoNT/C-LC-FLAG (wt) or pRS424-TEFpr-BoNT/C^{E230Q}-LC-FLAG (mut). Left panel: the cells were lysed and subjected to western blot analysis using anti-FLAG (BoNT/C-LC) and -actin antibodies. Right panel: Relative intensities of wild-type and mutant BoNT/C-LCs. (**b**) *spo20*Δ cells harboring Spo20/SNAP25(C)-3HA were transformed with pRS424-TEFpr (vector), pRS424-TEFpr-BoNT/A-LC-FLAG (wt), or pRS424-TEFpr-BoNT/A^{E224Q}-LC-FLAG (mut). Left panel: cell lysates were subjected to western blot analysis using anti-FLAG (BoNT/A-LC) and -actin antibodies. Right panel: Relative intensities of wild-type and mutant BoNT/A-LCs. (**c**) *spo20*Δ cells harboring HA-Spo20/SNAP25(C) were transformed with pRS424-SPO20pr (vector), pRS424-SPO20pr-BoNT/E1-LC-FLAG (wt), or pRS424-SPO20pr-BoNT/$E1^{E213Q}$-LC-FLAG (mut). Left panel: The cell lysates were subjected to western blot analysis using anti-FLAG (BoNT/E1-LC) and -actin antibodies. Right panel: Relative intensities of wild-type and mutant BoNT/E1-LCs. (**d**) *spo20*Δ cells harboring Spo20/SNAP25(C)-3HA were transformed with pRS424-SPO20pr (vector), pRS424-SPO20pr-BoNT/E12-LC-FLAG (wt), or pRS424-SPO20pr-BoNT/$E12^{E213Q}$-LC-FLAG (mut). Left panel: The cell lysates were subjected to western blot analysis using anti-FLAG (BoNT/E12-LC) and -actin antibodies. Right panel: Relative intensities of wild-type and mutant BoNT/E12-LCs. (**e**) *spo20*Δ cells harboring Spo20/SNAP25(N)-3HA were transformed with pRS424-SPO20pr (vector), pRS424-SPO20pr-BoNT/En-LC-FLAG (wt), or pRS424-SPO20pr-BoNT/En^{E226Q}-LC-FLAG (mut). Left panel: The cell lysates were subjected to western blot analysis using anti-FLAG (BoNT/En-LC) and -actin antibodies. Right panel: Relative intensities of wild-type and mutant BoNT/En-LCs. Intensities of BoNT-LC bands were normalized to actin. Intensity of wild-type BoNT-LC band was defined as 1. Data are presented as the mean ± SEM. Statistical significance was determined by two-tailed unpaired Student's *t* tests. n=3. **, $P < 0.01$; ****, $P < 0.0001$; ns, not significant ($P \geq 0.05$).

Figure S4

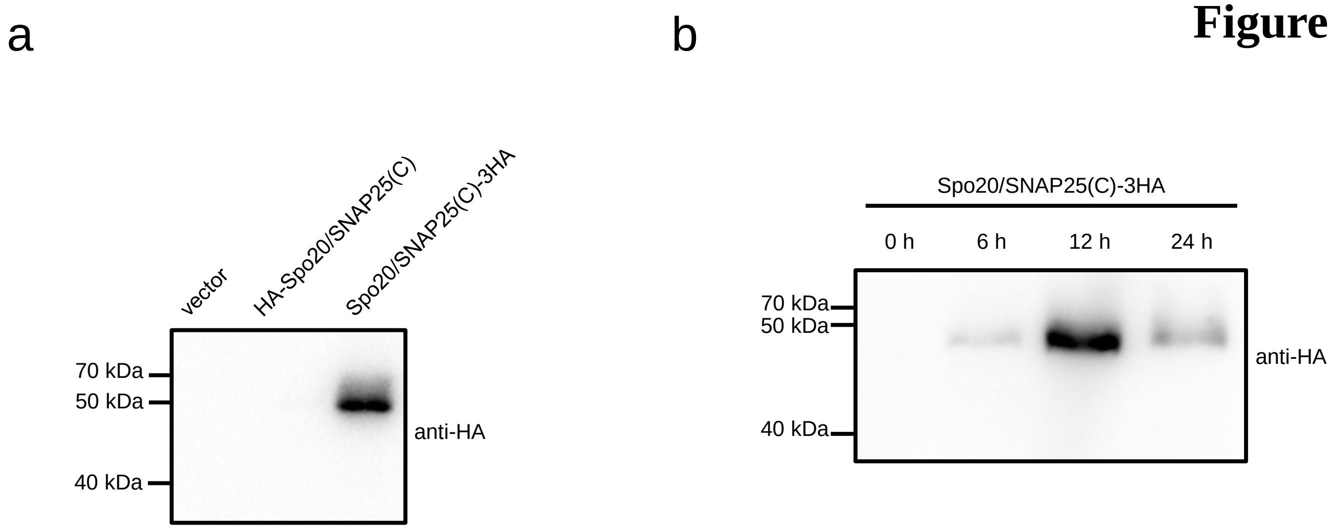

Figure S4 Detection of HA fusion proteins to Spo20/SNAP25(C) chimera.
(**a**) *spo20*Δ cells transformed with pRS306-SPO20pr (vector), pRS306-SPO20pr-HA-Spo20/SNAP25(C) (HA-Spo20/SNAP25(C)), or pRS426-SPO20pr-Spo20/SNAP25(C)-3HA (Spo20/SNAP25(C)-3HA) were cultured in sporulation medium for 12 h, and their lysates were subjected to western blot analysis using an anti-HA antibody. (**b**) *spo20*Δ cells were transformed with pRS426-SPO20pr-Spo20/SNAP25(C)-3HA (Spo20/SNAP25(C)-3HA) and the cells were cultured in sporulation media for indicated times. Their lysates were subjected to western blot analysis using an anti-HA antibody.

Figure S5

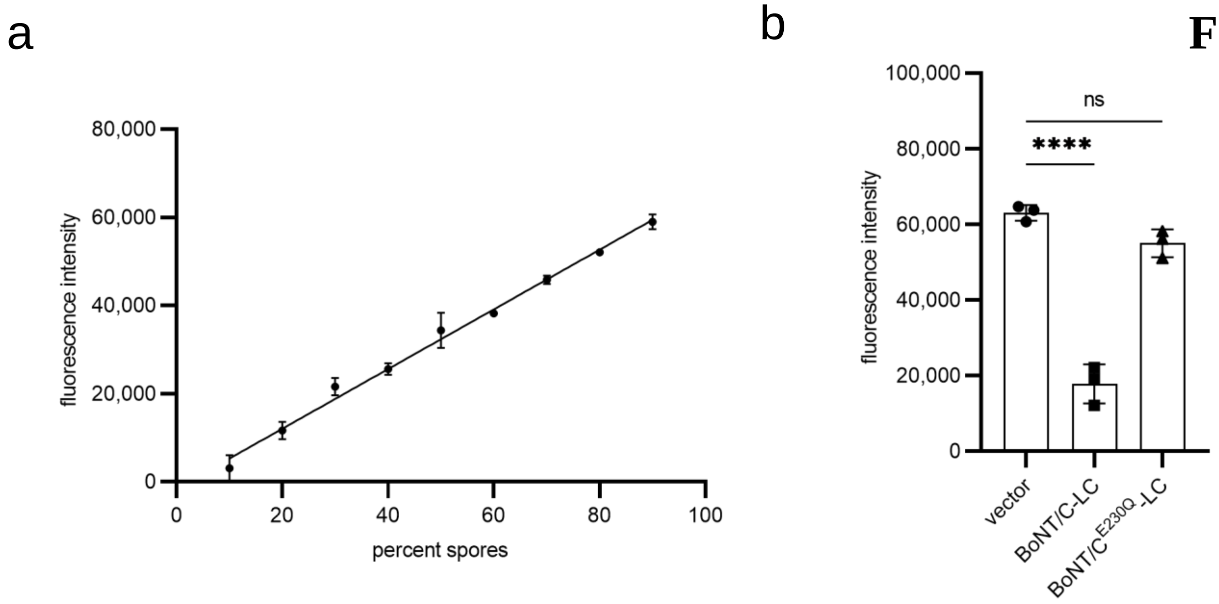

Figure S5 Colorimetric assay to measure sporulation efficiency.
(**a**) Spores of *spo20*Δ cells harboring pRS306-SPO20pr-HA-SPO20 were mixed with cells failed to produce spores and cell suspensions with various spore concentrations were prepared. Cells failed to produce spores were prepared by incubation of pRS306-SPO20pr-harboring *spo20*Δ cells in sporulation media. Fluorescent levels were measured with a microplate reader at an excitation wavelength of 285 nm and an emission wavelength of 425 nm. (**b**) *spo20*Δ cells harboring HA-Spo20/SNAP25(C) were transformed with pRS424-TEFpr (vector), pRS424-TEFpr-BoNT/C-LC-FLAG (wt) or pRS424-TEFpr-BoNT/C^{E230Q}-LC-FLAG (mut). They were incubated in sporulation media for 48 h. Sporulation efficiencies were determined with the colorimetric assay. Data are presented as the mean ± SEM. Statistical significance was determined by two-tailed unpaired Student's *t* tests. *n*=3. ****, $P < 0.0001$; ns, not significant ($P \geq 0.05$).

Figure S6

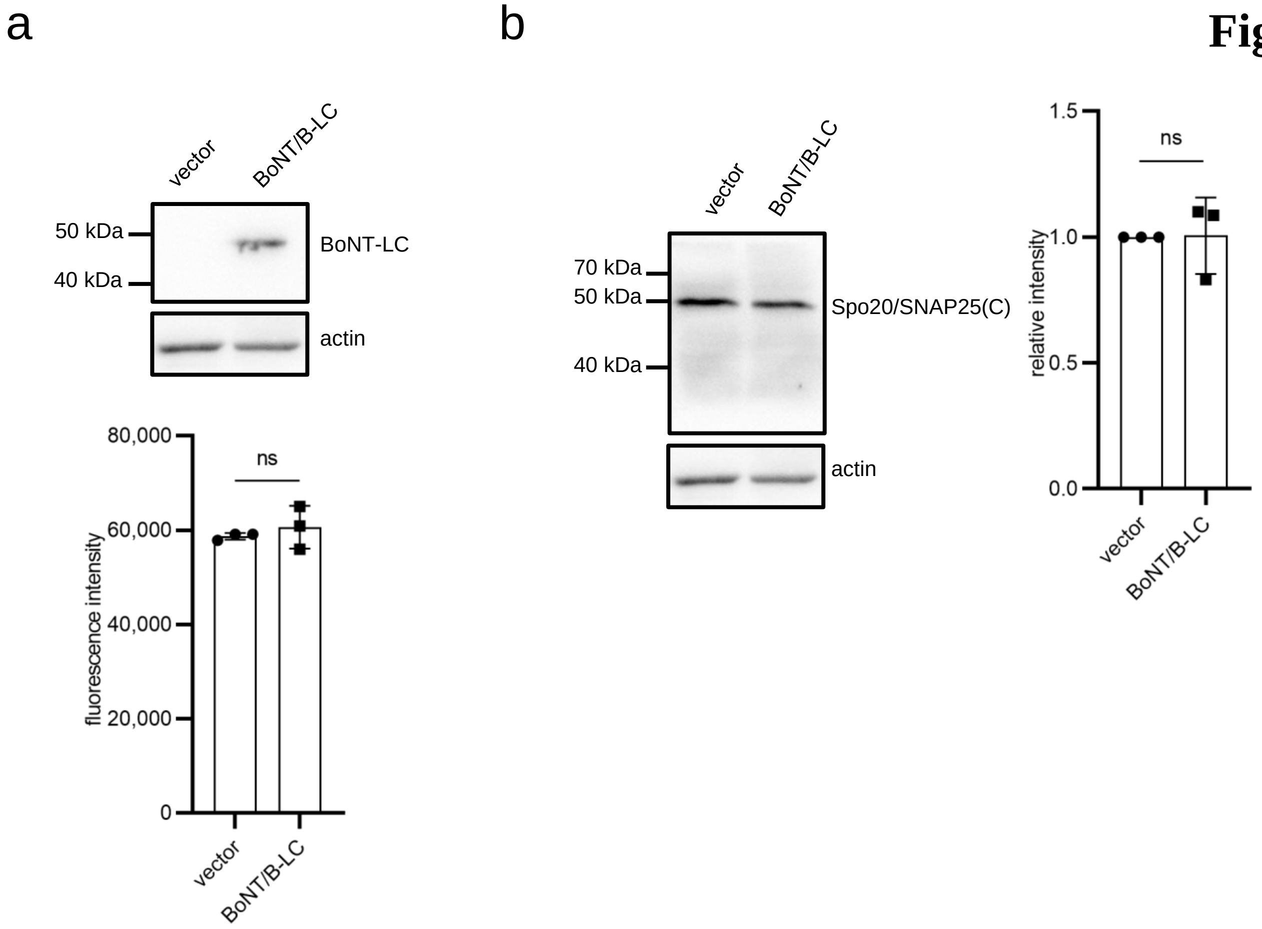

Figure S6 Expression of BoNT/B-LC in yeast cells harboring Spo20/SNAP25(C) chimera.
(**a**) *spo20*Δ cells harboring HA-Spo20/SNAP25(C) were transformed with pRS424-TEFpr (vector) or pRS424-TEFpr-BoNT/B-LC-FLAG (BoNT/B-LC). Top panel: The cell lysates were subjected to western blot analysis using anti-FLAG (BoNT-LC) and -actin antibodies. Bottom panel: The cells were incubated in sporulation medium for 48 h and sporulation efficiencies were measured with the colorimetric assay. (**b**) *spo20*Δ cells harboring Spo20/SNAP25(C)-3HA were transformed with pRS424-TEFpr (vector) or pRS424-TEFpr-BoNT/B-LC-FLAG (BoNT/B-LC). Left panel: The cell lysates were subjected to western blot analysis using anti-HA (Spo20/SNAP25(C)) and -actin antibodies. Right panel: Relative intensities of Spo20/SNAP25(C)-3HA detected in the cell lysates. Intensities of the Spo20/SNAP25(C)-3HA band were normalized to actin. Intensity of the band detected in the cells harboring pRS424-TEFpr was defined as 1. Data are presented as the mean ± SEM. Statistical significance was determined by two-tailed unpaired Student's *t* tests. *n*=3. ns, not significant ($P \geq 0.05$).

Figure S7

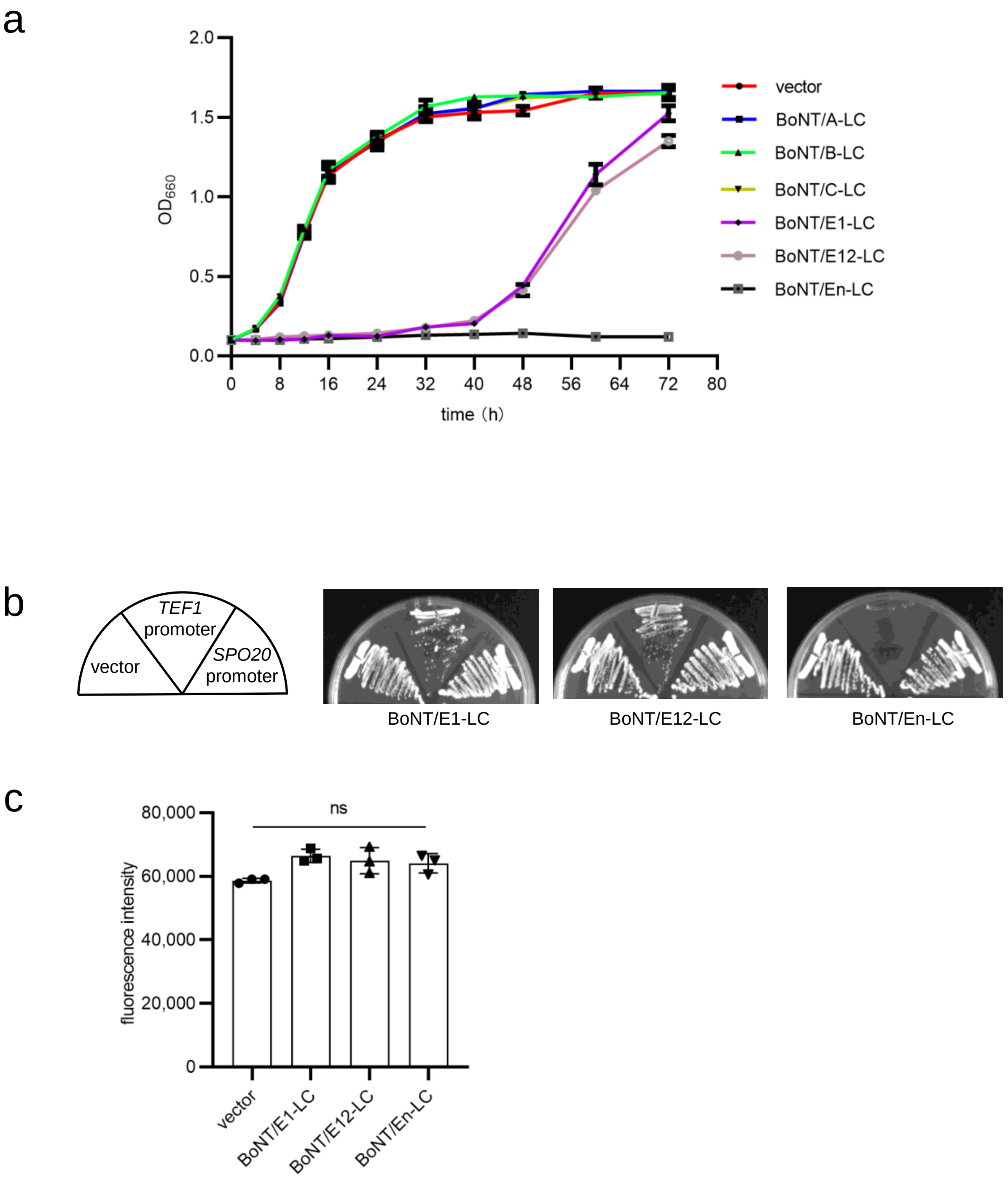

Figure S7 Effects of expression of BoNT/E1-LC, /E12-LC, and /En-LC in yeast cells.
(**a**) Wild-type cells harboring pRS424-TEFpr (vector), pRS424-TEFpr-BoNT/A-LC-FLAG (BoNT/A-LC), pRS424-TEFpr-BoNT/B-LC-FLAG (BoNT/B-LC), pRS424-TEFpr-BoNT/C-LC-FLAG (BoNT/C-LC), pRS424-TEFpr-BoNT/E1-LC-FLAG (BoNT/E1-LC), pRS424-TEFpr-BoNT/E12-LC-FLAG (BoNT/E12-LC), or pRS424-TEFpr-BoNT/En-LC-FLAG (BoNT/En-LC) were cultured in SD liquid media and growth curves were drawn. (**b**) In wild-type cells, BoNT/E1-LC, BoNT/E12-LC, or BoNT/En-LC were expressed under the control of constitutive *TEF1* promoter or sporulation specific *SPO20* promoter. The cells harboring pRS424-SPO20pr (vector) was used as a control. The cells were streaked onto SD plates and cultured for two days. (**c**) *spo20Δ* cells harboring HA-Spo20 were transformed with pRS424-SPO20pr (vector), pRS424-SPO20pr-BoNT/E1-LC-FLAG (BoNT/E1-LC), pRS424-SPO20pr-BoNT/E12-LC-FLAG (BoNT/E12-LC), or pRS424-SPO20pr-BoNT/En-LC-FLAG (BoNT/En-LC). The cells were cultured in sporulation medium for 48 h and sporulation efficiencies were measured with the colorimetric assay. Data are presented as the mean ± SEM (**a**, **c**). Statistical significance was determined by two-tailed unpaired Student’s *t* tests (C). *n*=3 (**a**, **c**). ns, not significant ($P \geq 0.05$).

Table S1 Plasmids used in this study.

Plasmid	Description	Source
pRS316TEF-SEC9	*URA3*, *CEN*, for expression of *SEC9* from constitutive *TEF1* promoter	This study
pRS314TEF	*TRP1*, *CEN*, empty vector with constitutive *TEF1* promoter	[39]
pRS314TEF-SNAP25	*TRP1*, *CEN*, for expression of *SNAP25* from constitutive *TEF1* promoter	This study
pRS314TEF-FLAG-SNAP25	*TRP1*, *CEN*, for expression of FLAG-*SNAP25* from constitutive *TEF1* promoter	This study
pRS314TEF-SEC9	*TRP1*, *CEN*, for expression of *SEC9* from constitutive *TEF1* promoter	This study
pRS314TEF-Sec9/SNAP25(C)	*TRP1*, *CEN*, for expression of Sec9/SNAP25(C) gene from constitutive *TEF1* promoter	This study
pRS314TEF-C1	*TRP1*, *CEN*, for expression of C1 gene from constitutive *TEF1* promoter	This study
pRS314TEF-C2	*TRP1*, *CEN*, for expression of C2 gene from constitutive *TEF1* promoter	This study
pRS314TEF-C3	*TRP1*, *CEN*, for expression of C3 gene from constitutive *TEF1* promoter	This study
pRS314TEF-C4	*TRP1*, *CEN*, for expression of C4 gene from constitutive *TEF1* promoter	This study
pRS314TEF-SGFP2	*TRP1*, *CEN*, for expression of SGFP2 gene from constitutive *TEF1* promoter	This study
pRS314TEF-SGFP2-SEC9	*TRP1*, *CEN*, for expression of SGFP2-*SEC9* from constitutive *TEF1* promoter	This study
pRS314TEF-SGFP2-Sec9/SNAP25(C)	*TRP1*, *CEN*, for expression of SGFP2-Sec9/SNAP25(C) gene from constitutive *TEF1* promoter	This study
pRS306-SPO20pr-HA-SPO20	*URA3*, *CEN*, integration, for expression of HA-*SPO20* from *SPO20* promoter	This study
pRS306-SPO20pr-HA-Spo20/SNAP25(N)	*URA3*, *CEN*, integration, for expression of HA-Spo20/SNAP25(N) gene from *SPO20* promoter	This study
pRS306-SPO20pr-HA-Spo20/SNAP25(C)	*URA3*, *CEN*, integration, for expression of HA-Spo20/SNAP25(C) gene from *SPO20* promoter	This study
pRS306-SPO20pr-HA-Spo20/SNAP25(NC)	*URA3*, *CEN*, integration, for expression of HA-Spo20/SNAP25(NC) gene from *SPO20* promoter	This study
pRS426-SPO20pr-SPO20-3HA	*URA3*, 2-µm, for expression of *SPO20*-3HA from *SPO20* promoter	This study
pRS426-SPO20pr-Spo20/SNAP25(N)-3HA	*URA3*, 2-µm, for expression of Spo20/SNAP25(N)-3HA gene from *SPO20* promoter	This study
pRS426-SPO20pr-Spo20/SNAP25(C)-3HA	*URA3*, 2-µm, for expression of Spo20/SNAP25(C)-3HA gene from *SPO20* promoter	This study
pRS426-SPO20pr-Spo20/SNAP25(NC)-3HA	*URA3*, 2-µm, for expression of Spo20/SNAP25(NC)-3HA gene from *SPO20* promoter	This study
pRS424TEF	*TRP1*, 2-µm, empty vector with constitutive *TEF1* promoter	[36]
pRS424TEF-BoNT/A-LC-FLAG	*TRP1*, 2-µm, for expression of BoNT/A-LC-FLAG (aa 1-448) gene from constitutive *TEF1* promoter	This study
pRS424TEF- BoNT/A^{E224Q}-LC-FLAG	*TRP1*, 2-µm, for expression of BoNT/A^{E224Q}-LC-FLAG gene from constitutive *TEF1* promoter	This study
pRS424TEF-BoNT/B-LC-FLAG	*TRP1*, 2-µm, for expression of BoNT/B-LC-FLAG gene (aa 1-441) from constitutive *TEF1* promoter	This study
pRS424TEF-BoNT/C-LC-FLAG	*TRP1*, 2-µm, for expression of BoNT/C-LC-FLAG (aa 1-449) gene from constitutive *TEF1* promoter	This study
pRS424TEF- BoNT/C^{E230Q}-LC-FLAG	*TRP1*, 2-µm, for expression of BoNT/C^{E230Q}-LC-FLAG gene from constitutive *TEF1* promoter	This study
pRS424-SPO20pr	*TRP1*, 2-µm, empty vector with *SPO20* promoter	This study
pRS424-SPO20pr-BoNT/E1-LC-FLAG	*TRP1*, 2-µm, for expression of BoNT/E1-LC-FLAG (aa 1-422) gene from *SPO20* promoter	This study
pRS424-SPO20pr- BoNT/E1^{E213Q}-LC-FLAG	*TRP1*, 2-µm, for expression of BoNT/E1^{E213Q}-LC-FLAG gene from *SPO20* promoter	This study
pRS424-SPO20pr-BoNT/E12-LC-FLAG	*TRP1*, 2-µm, for expression of BoNT/E12-LC-FLAG (aa 1-422) gene from *SPO20* promoter	This study
pRS424-SPO20pr- BoNT/E12^{E213Q}-LC-FLAG	*TRP1*, 2-µm, for expression of BoNT/E12^{E213Q}-LC-FLAG gene from *SPO20* promoter	This study
pRS424-SPO20pr-BoNT/En-LC-FLAG	*TRP1*, 2-µm, for expression of BoNT/En-LC-FLAG (aa 1-434) gene from *SPO20* promoter	This study
pRS424-SPO20pr- BoNT/En^{E226Q}-LC-FLAG	*TRP1*, 2-µm, for expression of BoNT/En^{E226Q}-LC-FLAG gene from *SPO20* promoter	This study

Table S2 Oligo nucleotides used in this study.

Name	Sequence
CSL1	TGAACCTAGCCTGGAGTAACTCGGAATAATACACCATTGACACCAAAAGACGGATCCCCGGGTTAATTAA
CSL2	GATGAAATGTTGTTACTTGTGATATATTACATAGTTTTTACAGGCATATTGAATTCGAGCTCGTTTAAAC
CSL3	TCAGCAATTTGGCTCTTCAG
CSL4	CTATTGCGTGCCCGAATGGT
CSL5	GGATCCATGGGATTAAAGAAATTTTT
CSL6	GTAAAGAATTAGACTCTCAGAATCGCCAGATCGACAGGAT
CSL7	GCGCCTCGAGCTAACCACTTCCCAGCATCTTTGTTGCACGTT
CSL8	GCGCCTCGAGTTAACCACTTCCCAGCATCTTTGTTGCACGTTGGTTGGCCTCATCAATTCTGGTTTTGTTGGAATCAGTGCTTTCC TCAATGTTGT
CSL9	GCGCCTCGAGTTAACCACTTCCCAGCATCTTTGTTGCACGTTGGTTGGCCTCATCAATATTGATATCTAGATCATCAG
CSL10	GCGCCTCGAGCTAACCACTTCCCAGCATCTTTGTTGCACGTTGGGTATTCATATGGAGATTGA
CSL11	ACACCTCGAGCTATCTGATACCTGCCAACCTGT
CSL12	TGGAATTGGAAATTGATAGGAACCTAGAGCAGGTGAGCGG
CSL13	CCGCTCACCTGCTCTAGGTTCCTATCAATTTCCAATTCCA
CSL15	CTCGAGTTAACCACTTCCCAGCATCT
CSL16	ACTAGTATGGACTACAAGGACGACGATGACAAGATGGCCGAAGACGCAGACAT
CSL17	ACTAGTATGGCCGAAGACGCAGACAT
CSL23	ACTAGTATGGTGAGCAAGGGCGAGGAG
CSL24	GGATCCTTACTTGTACAGCTCGTCC
CSL26	GTAAAGAATTAGACTCTCAGAATCGCCAGATCGACAGGAT
HXO26	TTTATTACTTTAGATTTTTCCGTTTGTGAATAGCCATTTTTA GATATATACGGATCCCCGGGTTAATTAA
HXO27	AAACTATTTATTCAATATATTTATACACGATATTTTGTGTGTA TAACAGAGAATTCGAGCTCGTTTAAAC
CSL28	CTCGAGTTAACCAGCCTTGTCATCGTCGTCCTTGTAGTCAGCACCAGCACCAGCACCTTTATTATAACCTTTATCCA
HXO28	ACCACAGTACGGAAAATACG
CSL29	GCGCCTCGAGTTAACCAGCCTTGTCATCGTCGTCCTTGTAGTCTTTATTATACAAAGATCTAC
HXO29	CTTTCGCAAGAATATCTGCG
CSL30	GAGCTCCGAAGGTGACGTAGCAATCC
CSL31	TCTAGATATATATCTAAAAATGGCTA
CSL32	CTCGAGTCTGTTATACACACAAAATA
CSL33	GGTACCTCATGAATGGAACGGGAAAC
CSL34	GCGCTCTAGATATATAATGTACCCATATGATGTTCCTGATTATGCTGGTGCAGGAGGGTTCAGAAAAATACTTGC
CSL35	GCGCCTCGAGTCACCATCTTTTCCCGATCA
CSL35-1	CTAAATTAAAATCTGTAAAAAGCACCCGTCGTATGCTGCA
CSL35-2	TGCAGCATACGACGGGTGCTTTTTACAGATTTTAATTTAG
CSL36-1	AGAATTTGACGGACCTAGGACATGAAAATAGAAGCCTGCT
CSL36-2	GGTTGGGTGATTTTAATGCAAGCAGGCTTCTATTTTCATGTCCTAGGTCCGTCAAATTCT
CSL37-1	TGGAAATCGACTTGTACGGAAACCTAGAACAAGTGAGCGG
CSL37-2	CCGCTCACTTGTTCTAGGTTTCCGTACAAGTCGATTTCCA
CSL38	GCGCCTCGAGTTAGCCACTTCCCAGCATCT
CSL40	ACTAGTATGCCATTTGTTAATAAACAATT
CSL41	ACTAGTATGCCAGTTACTATTAATAATTT
CSL42	ACTAGTATGCCAGTTACTATTAATAATTT
CSL44	GCGCTCTAGAATGGGGTTCAGAAAAATACT

Table S2 *Cont*.

CSL45	TCAGCACTGAGCAGCGTAATCTGGAACGTCATATGGATAGGATCCTGCATAGTCCGGGACGTCATAGGGATAGCCCGCATAGTCA GGAACATCGTATGGGTACTCGAGCCATCTTTTCCC
CSL46	TCAGCACTGAGCAGCGTAATCTGGAACGTCATATGGATAGGATCCTGCATAGTCCGGGACGTCATAGGGATAGCCCGCATAGTCA GGAACATCGTATGGGTACTCGAGGCCACTTCCCAG
CSL51	GCGCCTCGAGTTAACCAGCCTTGTCATCGTCGTCCTTGTAGTCTCTAACAGATTTACACATTT
CSL52	CAGCTGTTACTTTGGCTCATCAATTGATTCATGCTGGTCATAG
CSL53	CTATGACCAGCATGAATCAATTGATGAGCCAAAGTAACAGCTG
CSL54	CTCGAGCTCGAGTTACCCCGCCTTATCGTCATCGTCCTTGTAGTCCGCGCCCGCGCCTGCGCCACCTCTCTGTGAACT
CSL55	ACTAGTACTAGTATGGTAACAATCAA
CSL56	ACTAGTATGCCTAAGATTAACAGCTT
CSL57	CTCGAGTTAACCAGCTTTGTCGTCATCATCTTTGTAATCAGCTCCAGCACCGGCCCCCCTAATGCCCTTCACAGAGAC
CSL58	ACTAGTATGCCAAAGATCAACTCTTT
CSL59	CTCGAGTTACTTATCATCATCATCTTTATAATCTGCACCAGCACCAGCACCCCTAATACCCTTAACAG
CSL65	CAGCTTTAACTTTGATGCACGAATTAATCCACTCCTTACATGG
CSL66	CCATGTAAGGAGTGGATTAATTCGTGCATCAAAGTTAAAGCTG
CSL72	CTGCAGTTACGCTGATACACCAACTGTGCCACGGTCTACACGC
CSL73	GCGTGTAGACCGTGGCACAGTTGGTGTATCAGCGTAACTGCAG
CSL74	CAATTTTGATTTTGATGCATCAATTGAATCATGCTATGCATAA
CSL75	TTATGCATAGCATGATTCAATTGATGCATCAAAATCAAAATTG
CSL98	CCGCACTGACACTAATGCACCAATTAATACATAGCTTACATGG
CSL99	CCATGTAAGCTATGTATTAATTGGTGCATTAGTGTCAGTGCGG

Table S3 Strains used in this study.

Strain source	Genotype	Source
AN120	*MATα/MATa ARG4/arg4-NspI his3ΔSK/his3ΔSK ho::LYS2/ho::LYS2 leu2/leu2 lys2/lys2 RME1/rme1::LEU2 trp1::hisG/trp1::hisG ura3/ura3*	[28]
AN117-4B	*MATα ura3 leu2 trp1 hisΔSK arg4-NspI lys2 ho::LYS2 rme1::LEU2*	[28]
AN117-16D	*MATα ura3 leu2 trp1 hisΔSK lys2 ho::LYS2*	[28]
HW410	*MATα/MATa ARG4/arg4-NspI his3ΔSK/his3ΔSK ho::LYS2/ho::LYS2 leu2/leu2 lys2/lys2 RME1/rme1::LEU2 trp1::hisG/trp1::hisG ura3/ura3 spo20Δhis5*$^{+}$*/spo20Δhis5*$^{+}$	This study
YPH499	*MATa ura3-52 lys2-801_amber ade2-101_ochre trp1-Δ63 his3- Δ200 leu2-Δ1*	[29]

Supplementary Methods

Plasmids

The pRS316TEF-SEC9 and pRS314TEF-SEC9 plasmids were used to express *SEC9* from the *TEF1* promoter. The *SEC9* gene was amplified by PCR using primers CSL5 and CSL11. The yeast genomic DNA obtained from strain YPH499 [29] was used as a template. The PCR fragment was digested with *Bam*HI and *Xho*I and ligated into the similarly digested pRS316TEF and pRS314TEF [38]. Sec9/SNAP25(C) and other chimeric genes were also cloned into the *Bam*HI/*Xho*I sites of pRS314TEF. To obtain the Sec9/SNAP25(C) PCR product, fusion PCR was performed. First, the SNAP25 SNARE domain was amplified by PCR using the primers CSL11 and CSL12. Human brain cDNA library (TakaRa, China) was used as a template. Second, the *SEC9* gene was amplified by PCR using the primers CSL5 and CSL13 and pRS314TEF-SEC9 as a template. Third, fusion PCR was performed with the two PCR products and primers CSL5 and CSL11. C1 chimera was constructed similarly. Primers used to generate this chimera are CSL5, CSL26, CSL6 and CSL7. To construct C2, C3, and C4 chimeras, PCR was performed with primers CSL5 and CSL8 (C2), CSL5 and CSL9 (C3), and CSL5 and CSL10 (C4). pRS314TEF-SEC9 was used as a template. The pRS314TEF-SGFP2, -SGFP2-SEC9 and -SGFP2-Sec9/SNAP25(C) plasmids were used to express strongly enhanced green fluorescent protein (SGFP2) [39] fusions. To construct these plasmids, SGFP2 gene without the stop codon was amplified by PCR using primers CSL23 and CSL24 and pRS314-SSO1pr-SGFP2-STX1A [35] as a template. The PCR fragment was digested with *Spe*I and *Bam*HI and ligated into similarly digested pRS314TEF, pRS314TEF-SEC9 and pRS314TEF-Sec9/SNAP25(C).

The pRS314TEF-FLAG-SNAP25 plasmid was used to express SNAP25 fused to a FLAG tag at the 5' terminus from the *TEF1* promoter. To construct this plasmid, the FLAG-SNAP25 gene was amplified by PCR using the primers CSL15 and CSL16. Human brain cDNA library (TaKaRa) was used as a template. The PCR fragment was ligated into the *Spe*I/*Xho*I sites of pRS314TEF. The pRS314TEF-SNAP25 plasmid was used to express SNAP25 from the *TEF1* promoter. To construct this plasmid, the SNAP25 gene was amplified by PCR using the primers CSL16 and CSL17. pRS314TEF-FLAG-SNAP25 was used as a template. The PCR fragment was ligated into the *Spe*I/*Xho*I sites of pRS314TEF.

The pRS306SPO20pr-HA-SPO20 plasmid was used to express *SPO20* fused to a HA tag at the 5' terminus from the *SPO20* promoter. To construct this plasmid, the *SPO20* promoter and terminator were amplified by PCR using the primers CSL30 and CSL31, and CSL32 and CSL33, respectively. Yeast genomic DNA obtained from strain AN120 [28] was used as a template. These PCR fragments were ligated into the *Sac*I/*Xba*I, and *Xho*I/*Kpn*I sites of pRS306 [29], respectively. The resulting plasmid was named pRS306-SPO20pr. The *SPO20* gene was amplified by PCR using the primers CSL34 and CSL35. Yeast genomic DNA obtained from strain AN120 was used as a template. The PCR fragment was cloned into the *Xba*I/*Xho*I sites of pRS306-SPO20pr. Spo20/SNAP25(N) and other chimeric genes were also cloned into the *Xba*I/*Xho*I sites of pRS306-SPO20pr. Spo20/SNAP25 chimeric SNAREs were generated by fusion PCR. pRS306-SPO20pr-HA-SPO20 and pRS306-SPO20pr-HA-Spo20/SNAP25(N) were used as templates for HA-Spo20/SNAP25(N) and HA-Spo20/SNAP25(C), and HA-Spo20/SNAP25(NC), respectively. To amplify SNARE domains of SNAP25, human brain cDNA (TakaRa) was used as a template. Primers used for the fusion PCR reactions were follows: CSL35-1, CSL36-1, CSL35-2, CSL35-2, and CSL34, CSL35 for HA-Spo20/SNAP25(N); CSL37-1, CSL37-2, CSL34 and CSL38 for HA-Spo20/SNAP25(C) and HA-Spo20/SNAP25(NC). The pRS426-SPO20pr-SPO20-3HA, -Spo20/SNAP25(N)-3HA, -Spo20/SNAP25(C)-3HA and -Spo20/SNAP25(NC)-3HA plasmids were used to express the corresponding genes fused to a 3 × HA tag at the 3′ terminus from the *SPO20* promoter. Primers used to amplify the 3 × HA fusion genes were follows: CSL44 and CSL45 for SPO20-3HA and Spo20/SNAP25(N)-3HA; CSL44 and CSL46 for Spo20/SNAP25(C)-3HA and Spo20/SNAP25(NC)-3HA. The pRS306-SPO20pr-HA-SPO20, -HA-Spo20/SNAP25(N), -HA-Spo20/SNAP25(C) and -HA-Spo20/SNAP25(NC) plasmids were used as the templates, respectively. The PCR fragments were ligated into the *Xba*I/*Xho*I sites of pRS306-SPO20pr. Then, pRS306-SPO20pr-SPO20-3HA, pRS306-SPO20pr-Spo20/SNAP25(N)-3HA, pRS306-SPO20pr-Spo20/SNAP25(C)-3HA and pRS306-SPO20pr-Spo20/SNAP25(NC)-3HA plasmids were digested with *Sac*I/*Kpn*I and the fragments were ligated into similarly digested pRS426.

The pRS424TEF-BoNT/A-LC-FLAG, -BoNT/B-LC-FLAG and -BoNT/C-LC-FLAG plasmids were used to express BoNT/A-LC (amino acids 1-448), BoNT/B-LC (amino acids 1-441), BoNT/C-LC (amino acids 1-449) fused to a FLAG tag at the 3′ terminus from the *TEF1* promoter. The BoNT/A-LC, BoNT/B-LC and BoNT/C-LC encoding genes were synthesized by GENEWIZ (China). Using the synthesized BoNT/A-LC, BoNT/B-LC and BoNT/C-LC as templates, BoNT/A-LC-FLAG, BoNT/B-LC-FLAG and BoNT/C-LC-FLAG genes were amplified by PCR with the primers CSL40 and CSL28, CSL42 and CSL51, CSL41 and CSL29, respectively. The fragments were cloned into the *Spe*I/*Xho*I sites of pRS426-TEF [38]. The pRS424-SPO20pr-BoNT/En-LC-FLAG, -BoNT/E1-LC-FLAG, and -BoNT/E12-LC-FLAG plasmids were used to express BoNT/En-LC (amino acids 1-434), BoNT/E1-LC (amino acids 1-422) and BoNT/E12-LC (amino acids 1-442) fused to a FLAG tag at the 3′ terminus of the *SPO20* promoter. To construct pRS424-SPO20pr plasmid, pRS306-SPO20pr was digested with *Sac*I/*Xba*I and the fragment containing the SPO20 promoter was ligated into similarly digested pRS424TEF-CYC1ter. The BoNT/En-LC, BoNT/E1-LC and BoNT/E12-LC genes were synthesized by GENEWIZ. Using the synthesized BoNT/En-LC, BoNT/E1-LC and BoNT/E12-LC as templates, BoNT/En-LC-FLAG, BoNT/E1-LC-FLAG, and BoNT/E12-LC-FLAG were amplified by PCR with the primers CSL55 and CSL54, CSL56 and CSL57, and CSL58 and CSL59, respectively. The fragments were then cloned into the *Spe*I/*Xho*I sites of pRS426-SPO20pr.

Site-directed mutations were introduced by PCR. The primers CSL52 and CSL53 were used to introduce the E224Q mutation into BoNT/A-LC; the primers CSL74 and CSL75 were used to introduce the E230Q mutation into BoNT/C-LC; the primers CSL72 and CSL73 were used to introduce the E226Q mutation into BoNT/En-LC; the primers CSL98 and CSL99, and CSL65 and CSL66 were used to introduce the E213Q mutation into BoNT/E1-LC and BoNT/E12-LC, respectively.
